# Agonistic experience during development establishes inter-individual differences in approach-avoidance behaviour of crickets

**DOI:** 10.1038/s41598-021-96201-1

**Published:** 2021-08-17

**Authors:** Julia S. Balsam, Paul A. Stevenson

**Affiliations:** Institute for Biology, Faculty of Life Sciences, Leipzig University, Talstr. 33, 04103 Leipzig, Germany

**Keywords:** Social behaviour, Animal behaviour

## Abstract

Members of numerous animal species show consistent inter-individual differences in behaviours, but the forces generating animal “personality” or individuality remain unclear. We show that experiences gathered solely from social conflict can establish consistent differences in the decision of male crickets to approach or avoid a stimulus directed at one antenna. Adults isolated for 48 h from a colony already exhibit behavioural differences. Prior to staging a single dyadic contest, prospective winners approached the stimulus whereas prospective losers turned away, as they did also after fighting. In contrast, adults raised as nymphs with adult males present but isolated from them as last instar nymphs, all showed avoidance. Furthermore, adults raised without prior adult contact, showed no preferred directional response. However, following a single fight, winners from both these groups showed approach and losers avoidance, but this difference lasted only one day. In contrast, after 6 successive wins or defeats, the different directional responses of multiple winners and losers remained consistent for at least 6 days. Correlation analysis revealed examples of consistent inter-individual differences in the direction and magnitude of turning responses, which also correlated with individual aggressiveness and motility. Together our data reveal that social subjugation, or lack thereof, during post-embryonic and early adult development forges individuality and supports the notion of a proactive–reactive syndrome in crickets.

## Introduction

One of the most influential recent findings in the field of animal behaviour is that individuals of the same species, including many invertebrates, exhibit consistent inter-individual differences in specific behavioural traits^1–4^, commonly referred to as “personality”, but here as “individuality”, a less anthropomorphic term. Furthermore, suites of different behavioural traits exhibited by individual animals are often found to correlate positively across time and different contexts forming so-called “behavioural syndromes”^3,5^. The proximate mechanisms generating behavioural individuality and syndromes in animals are, however, still poorly understood^4^. Data from across the animal kingdom suggest that individual differences in behaviour are nearly always influenced by genetic factors^6^. However, the occurrence of inter-individual behavioural differences in genetically homogeneous humans^7^ and invertebrates^8^ highlights “nurture” during development as being at least as important for some traits as “nature”. The factors driving animal individuality are thus currently considered to include potentially all aspects of an individual’s physiology and environment^3^, which can vary across species, developmental stages, context and situation^9^. Candidates include immune challenge^10,11^, differences in nutrient supply^12^, diet^13^, general body condition^14^, metabolic rate^15^ and aggressive experience^16^.

Much of the pioneering work on invertebrates has focused on adult crickets and documents that both free living and captive members of several species show repeatable inter-individual differences in traits such as aggressiveness, general locomotor activity and exploratory behaviour (*Acheta domesticus*^17,18^, *Gryllus bimaculatus*^19^, *Gryllus campestris*^20–23^, *Gryllus integer*^10,24^). It has also become apparent that dominant adult males, in addition to being more aggressive, also tend to be more active, more exploratory and more likely to approach a novel stimulus than subordinates (*G. bimaculatus*^19^, *G. campestris*^23^, *G. integer*^25^, *Teleogryllus oceanicus*^26^) and that changes in dominance status can erode existing differences (*Teleogryllus oceanicus*^26^). In the majority of studies, however, correlations between individual aggressiveness with other traits, required for verifying a “proactive–reactive” syndrome^5^, were not found (*Acheta domesticus*^17^, *G. bimaculatus*^19^, *G. integer*^27^; see however *G*. *campestris*^23^). Moreover, the extent to which early-life social experience during development is involved in actually establishing adult behavioural individuality, and therefore potentially also behavioural syndromes, remains unclear.

Notably, after finding that dominant *G. bimaculatus* males that won two consecutive dyadic contests in a knockout tournament are more active and exploratory than subordinates that lost two contests, the same traits were also found in the prospective winners and losers one day before the tournament^19^. Hence, the behavioural difference must be due to some earlier event in development. The involvement of prior aggressive experience seemed doubtful, since the animals were socially isolated for two days before testing, to negate the well-known effects of winning and losing on subsequent behaviour^28^, which at the time were known to last little more than 3 h in crickets^29,30^. It was established later, however, that multiple intermittent defeats lead to practically life-long depression of agonistic behaviour in adult crickets^31,32^. Furthermore, adult crickets raised isolated from adult males throughout post-embryonic development, become more aggressive and motile than adults raised together with adult males^33^ (see also for crayfish^34,35^ and cockroaches^36^). This suggests that differences in adult behaviour in crickets could arise from defeat stress during development, as shown in rodent models for human depression^37,38^, or alternatively as a more general consequence of deprivation of environmental stimulation during isolation^39,40^.

In this paper we test the hypothesis, that multiple winning and losing experiences during post-embryonic development and/or early adult life, establish consistent inter-individual differences in the behaviour of adult crickets. First, we employ automated video-tracking to evaluate the effects of isolation from adult males, for varying lengths of time during development, on the decision to approach or avoid a tactile stimulus applied to one antenna, using a severed antenna from another male. Since antennal stimulation can evoke aggressive behaviour when applied for about 2 s at 20 Hz, as occurring naturally during antennal fencing between males^41^, we applied only a single, brief (33–50 ms) stimulus to minimise this. We next tested whether the response was influenced by subsequent single and multiple winning or losing experiences. We speculate, that adults reared under standard, sex- and age-mixed laboratory conditions will differ in their behaviour. Specifically, we anticipate that some adults will turn towards the antennal stimulus, while others turn away, and that these differences will not be evident in animals raised isolated from adult males, but can be induced by experiencing multiple wins or defeats against other adult males. Our results support the hypothesis that early-life adversity, or lack thereof, generates consistent, inter-individual behavioural differences manifested in an aggressive-proactive, submissive-reactive behavioural syndrome.

## Methods

### Experimental animals and ethical note

All experiments were performed on sexually mature adult male Mediterranean field crickets, *Gryllus bimaculatus* (de Geer) that were bred and maintained at the animal housing facility of the University of Leipzig. These hemimetabolous insects pass through 8 nymphal stages, each lasting 4–5 days, before moulting to the adult, which are readily identified by the possession of functional hind-wings and hardened forewings^42^. They were kept in groups of 30–40 in transparent plastic boxes (35 × 19 cm, 30 cm high), with a sand covered floor and egg cartons for shelter, under constant standard conditions (22–24 °C, relative humidity 40–60%, 12 h: 12 h light: dark regime, daily feeding on bran, fresh carrots and water ad libitum). All experiments were performed in the months of April to October, at room temperature (20–24 °C) during daylight hours, excluding midday and generally rainy, overcast days when the general behaviour of our crickets tends to be subdued^43,44^. When the same animals were tested on consecutive days this was performed at approximately the same time of day (± 1 h). All treatments conformed to Principles of Laboratory Animal Care and German Law on Protection of Animals (*Deutsches Tierschutzgesetz*). Our analysis is based on observations of a total of 250 crickets.

### Experimental groups

**Figure 1:**
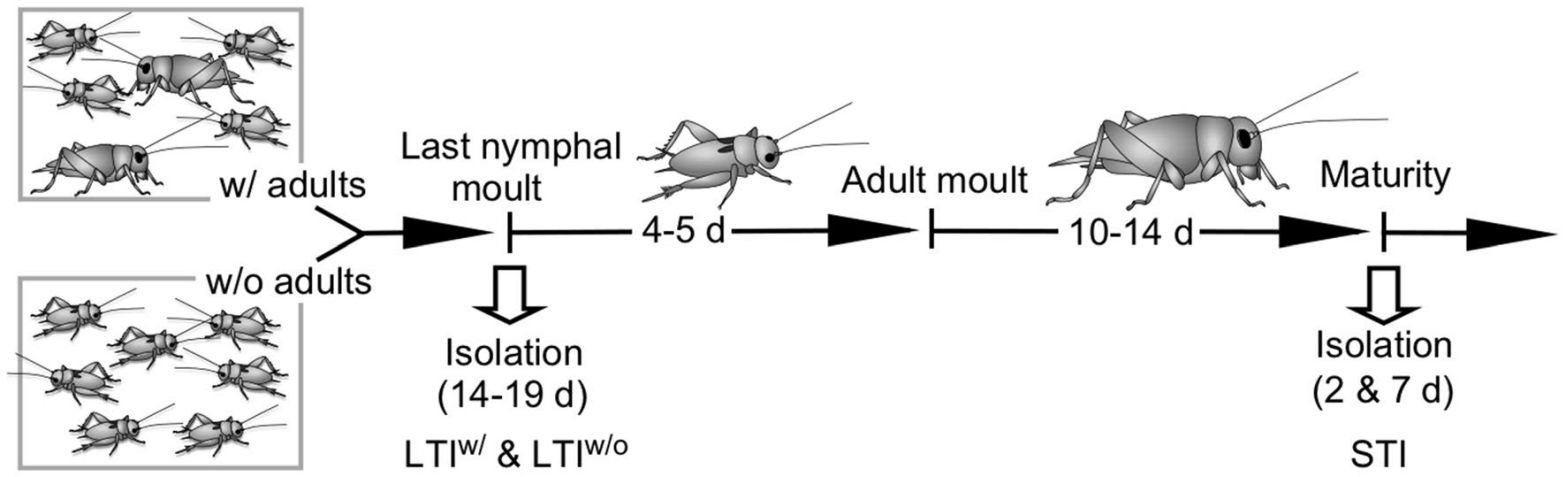
Schema illustrating key events of cricket life history together with culture conditions and periods of social isolation for the different experimental groups of adult male crickets. *LTI*^*w/*^: long term isolates with prior contact to adult males during nymphal life, *LTI*^*w/o*^: long term isolates without prior contact to adult males during nymphal life, *STI*: short term isolates, separated at maturity for 2 or 7 days. All components of the figures were created with canvas DRAW 5 for MAC (Version 5.0.2, ACD Systems, https://www.acdsystems.com/index).

Animals with differing social experiences were generated by varying the social composition of the colony and the duration of social isolation, when animals were kept in individual glass jars (400 cm^3^), under the same ambient conditions for different lengths of time until experimentation (Fig. 1).

#### STI: short term isolates with prior contact to adults as nymphs and adults

Here, males were taken from the colony as mature adults and kept socially isolated for 48 h in individual glass jars until experimentation. These animals thus had social contact to adults and nymphs of both sexes from hatching onwards, throughout all nymphal stages and as young adults until maturity, when they achieve their peak dominance, which occurs 10 to 14 days after the final moult^45^. An additional control group of animals (mentioned explicitly in results) was also isolated as mature adults but kept in isolation for 7 days until experimentation.

#### LTI^w/^: long term isolates with contact to adults as nymphs only

These were raised under the same social conditions as STI, but taken from the colony on the day of the moult to the last nymphal stage, identified by the size and shape of the wing-patches and when still pale in colour. They were kept individually in social isolation throughout the last nymphal stage (4–5 days) and also as young adults for 10 to 14 days until sexual maturity.

#### LTI^w/o^: long term isolates without prior contact to adults

These were raised in the complete absence of adults from hatching onwards. As for LTI^w/^, they were taken from the colony on the day of the moult to the last nymphal stage and kept individually in social isolation until adult maturity. These animals thus only had contact to nymphs during development, which do not interact aggressively to generate winners and losers with long-term consequences for their subsequent behaviour^33^.

### Turning response to antennal stimulation

The behaviour of focal crickets was observed in a round glass arena (diameter: 18 cm) with a filter paper-covered floor. A digital video camera (Basler acA1920-155uc, Ahrensburg, Germany, 60 frames/s) recorded the responses to touching one antenna briefly with a freshly excised antenna from another adult male. In our freely moving, unrestrained crickets this was most easily achieved manually, using an antenna attached to the distal end of a 20 cm long, white, wooden stick to minimise visual stimulation and detection by a video-tracking system. The operator was also obscured from the animal’s view by a white paper sheet. The animals were placed in the arena 1 min before experimentation, to allow them to adapt to the new surroundings. To avoid startling the animal, the severed antenna was positioned slowly in the vicinity of the animal’s head and held still for at least 1 s before stimulation. The stimulus was first applied when the animal was not excessively mobile and delivered only once to the most accessible antenna. To achieve this, the severed antenna was moved above the focal animal´s antenna, and a single touch was applied with a force just sufficient to deflect the antenna visibly (average duration of stimulus stroke: 133–200 ms, touch duration: 33–55 ms, as measured from the number of video frames). Pilot studies revealed no difference between left- and right-side antennal stimulation and no response to sham stimulation without a touch. Video-taped responses were stored on a computer (Dell Precision 3620, Round Rock, Texas, USA) and analysed with commercial video-tracking software (Noldus EthoVision XT, Version 14, Wageningen, Netherlands, https://www.noldus.com/ethovision-xt). Three-point tracking was employed to detect the body centre, head and abdominal tip (*centre*, *head* and *tail-base*) of each cricket by the grey scaling method (minimum grey value 0, maximum 140) to track the position of the animal’s longitudinal axis. To avoid continuous detection of minimal movements, for example from ventilation, we set a “minimum distance moved” filter of 0.02 cm, under which no response was recorded. We thus measured each animal’s angular turning response towards or away from the stimulus from the moment of touch, frame for frame, every 16.7 ms for a total of 1 s. Raw data of each track were adjusted to set the longitudinal axis of the animal’s starting position to 0° (Microsoft Excel for MAC, Version 15.23, Microsoft 2016, Redmond, WA, USA, https://www.microsoft.com/de-de/microsoft-365/excel). Angular changes > 90° in 1 frame, that occasionally occurred due to erroneous detection of the head and tail points during rapid movements, were replaced by the mean of the previous and subsequent measurement. In some experiments, we also recorded the total distance moved by each animal over a 3 min period in the same arena just before applying the antennal stimulus.

### Influence of dominance and subordination (winning and losing)

Contests were staged between weight-matched pairs (< 5% difference) of each experimental group by placing them at opposite ends of an arena (16 × 9 cm, 7 cm high) separated in the middle by a sliding door. On removing the door, the crickets engage in fights that generate clear losers and winners. Losers retreat and avoid other males^30,31^, whereas winners generate the characteristic rival song and remain aggressive towards other males^29^. To analyse the effects of social experience, we first measured turning responses in individual crickets before staging their first fight, when the animal’s social status as dominants or subordinates was unknown. After 1 h, contests were staged between pairs of males matched by weight alone, irrespective of their previous turn angle, in order to generate winners and losers. Turn angle was then measured again after fighting, for the winners and losers, and the performance of each individual was compared to its performance before fighting, in the prospective winners and prospective losers.

We also analysed the influence of multiple wins and defeats on turning responses. For this, pairs of crickets of similar weights were matched against each other 6 times in succession at 30 min intervals to yield sixfold winners and sixfold losers. In most cases, the winners of the first fight won all successive fights, if not the animals were not included in the analysis.

To test for correlation between turning and individual aggressiveness, we first recorded the turn angles of STI crickets of unknown social status and then matched them against a standard hyper-aggressive male generated by flying in a wind stream for 1 min^46^. All focal animals engaged the hyper-aggressive opponents, but retreated after 1–15 s and subsequently avoided further contact with the opponent, which signalled victory by generating the rival song and chasing the focal animal. Thus, since the focal animal always lost these contests, we measured their individual aggressive motivation by recording the total duration of their interaction with the hyper-aggressive winner from initial antennal contact to retreat with a stopwatch to the nearest second^33^.

### Data analysis

All statistical tests were performed using commercial software (GraphPad Prism 7 for MAC, Version 7.0c, GraphPad Software, Inc., La Jolla, CA, USA, https://www.graphpad.com) running on a personal computer (Apple, Cupertino, CA, USA). The Shapiro–Wilk test revealed our data sets to be normally distributed. Accordingly, we give for each data the means and 95% confidence interval (CI) in preference to the standard deviation^47^ and test for significant difference between the same groups before and after the fight or between different groups with Student´s two-tailed paired, respectively unpaired t-test. To test for correlations, we used Pearson´s correlation. Fisher´s exact test was performed to compare relative frequencies of behaviours. The number of crickets for each analysis is given in the legends. For single comparisons, the significance level alpha was set to *p* < 0.05. Occasionally, we used the same data sets in three tests and applied the Bonferroni correction to alpha (*p* < 0.017, mentioned in legends; for arguments and references on multiple comparisons see^19^).

## Results

### Short term isolates with adult contact as nymphs and adults (STI)

In response to the single touch stimulus, crickets tended to turn either towards it (positive turners) or away (negative turners). We never observed the aggressive mandible threat after the brief, single stimulus, which often occurs in response to repetitive stimulation^33,48^. However, positive turners (*n* = 40 observed) occasionally lunged forward (*n* = 9), exhibited body jerks (*n* = 11) or generated the rival song (*n* = 4), which was never shown by negative turners.

**Figure 2:**
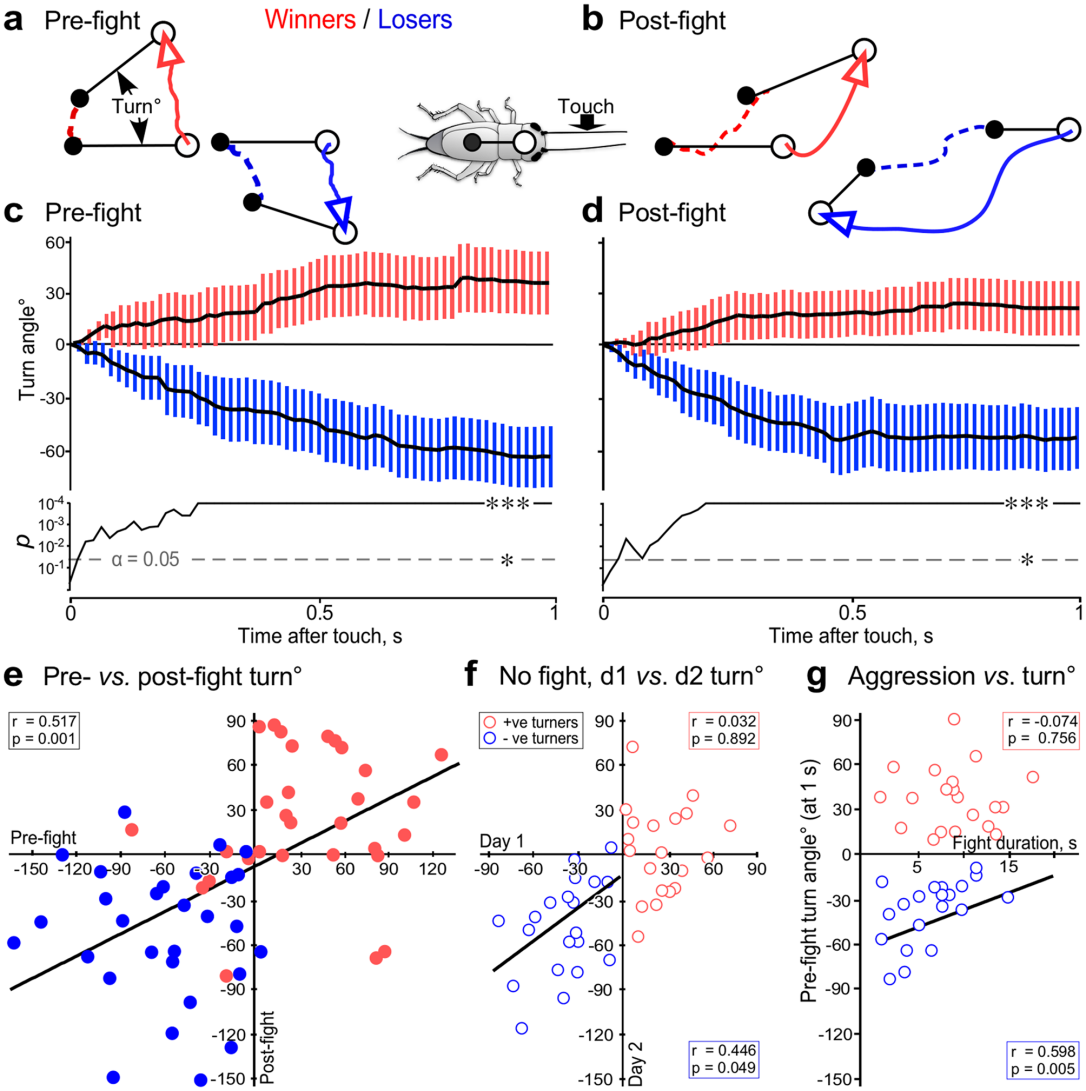
Turning responses and aggression in short term isolates (STI). (**a**) Original tracks of responses to the touch stimulus in a prospective winner and loser (red respectively blue arrows; black lines: body orientation; full circle: body; open circle: head). (**b**) Same animals as in (**a**) 1 h after their first fight. (**c**) Top graph: sequential plots of mean turn angles (black lines) for every frame with 95% CI (bars, red: prospective winners; blue: prospective losers; *n* = 30 each). Bottom graph: significance of difference *p* between prospective winners and losers, * *p* < 0.05, *** *p* < 0.001. (**d**) As for (**c**) 1 h after first fight. (**e**) Plots of pre-fight *versus* post-fight turning responses for data depicted in (**c,d**). (**f**) Plots of turn angles on two consecutive days without an interposing fight. Red and blue open circles indicate individuals showing positive (+ ve), respectively negative (−ve) turns at the first trial, *n* = 20 each. (**g**) Plots of fight duration against hyper-aggressive opponents *versus* pre-fight turn angle for positive and negative turners (red and blue open circles respectively, *n* = 20 each). All components of the figures were created with canvas DRAW 5 for MAC (Version 5.0.2, ACD Systems, https://www.acdsystems.com/index).

Figure 2a–d shows example traces and sequential plots of the turning responses of STI crickets before and after winning or losing a single aggressive encounter against a weight-matched male from the same experimental cohort. Before fighting, the crickets with still unknown social status turned either towards or away from the direction of the stimulated antenna (37% positive turn angle, respectively 58% negative turn angle, 5% no clear directional preference, *n* = 60). After fighting (Fig. 2d) the majority of the now established winners turned towards the stimulus (63%, *n* = 30) and losers away from it (77%, *n* = 30). A sequential plot of the difference-probability *p* revealed a significant difference between the mean turn angle for these groups 2 frames after the touch stimulus (Fig. 2d; 34 ms, *p* = 0.048). After 1 s, winners had turned + 22° and losers − 58° (means, 95% CI = 5 to 39 and − 78° to − 39° respectively, significantly different to winners: *p* < 0.001, *n* = 30 each). Retrospective sorting of the data revealed that before fighting, the majority of prospective winners also turned towards the stimulus (83%, *n* = 30), whereas the prospective losers mainly showed avoidance (90%, *n* = 30). The mean turn angle for prospective winners and prospective losers was significantly different to each other after 2 frames (34 ms, *p* = 0.008). When measured 1 s after the touch, the prospective winners had turned + 34° and the prospective losers −63° (means, 95% CI = 16 to 52 and −80 to −46 respectively, significantly different to prospective winners: *p* < 0.001, *n* = 30 each). Disregarding angular direction, losers on average made turns almost twice as large as winners (Fig. 2d), but this was not quite statistically significant (*p* = 0.050).

A plot of each individual’s turn angle before *versus* after the contest revealed a linear correlation for the entire data set (Fig. 2e; *r* = 0.517, *p* < 0.001, *n* = 60; Table 1). However, separate plots for winners and losers revealed no correlation (Fig. 2e; winners: *r* = 0.112, *p* = 0.557; losers: *r* = 0.087, *p* = 0.647, *n* = 30 each; Table 1). To check for influences of the interposing fight, we measured the initial response to the touch stimulus just after isolation and again on the following day in a separate cohort of STIs without staging a contest in between. A plot of each individual’s turn angle on day 1 *versus* day 2 revealed a linear correlation for negative turners but not for positive turners (Fig. 2f; positive tuners: *r* = 0.032, *p* = 0.892; negative turners: *r* = 0.446, *p* = 0.049, *n* = 20 each; Table 1). We then measured turning responses in another cohort of STIs and subsequently matched positive turners against negative turners in a dyadic contest. Positive turners won almost all the fights (90%, significantly different to negative turners: *p* < 0.001 and to 50%: *p* = 0.014, *n* = 20; not illustrated). For an additional cohort of STIs we evaluated the turning responses before fighting and subsequently measured the total time each individual spent engaging in a fight against a hyper-aggressive opponent before retreating (aggressive persistence). On average, positive turners persisted significantly longer than negative turners and were hence more aggressive (mean persistence: 9 s, respectively 6 s, *p* = 0.01, *n* = 20 each; not illustrated). Furthermore, for individuals showing negative turns in response to the touch stimulus, their turn angle correlated positively with persistence, but this was not evident for positive turners (Fig. 2g; positive tuners: *r* = − 0.074, *p* = 0.756; negative turners: *r* = 0.598, *p* = 0.005, *n* = 20 each; Table 1).

**Table 1:** Correlations between measured variables for selected test groups of individuals.

X-axis	Y-axis	Test group	*r*	*p*
Turn angle pre-fight	Turn angle post-fight	STI winners & losers	**0.517**	** < 0.001**
		STI winners	0.112	0.557
		STI losers	0.087	0.647
Turn angle day 1	Turn angle day 2	STI + ve & −ve turners	**0.661**	** < 0.001**
		STI + ve turners	0.032	0.892
		STI -ve turners	**0.446**	**0.049**
Turn angle	Aggression	STI + ve & −ve turners	**0.472**	**0.002**
		STI + ve turners	− 0.074	0.756
		STI −ve turners	**0.598**	**0.005**
Turn angle	Motility	STI + ve & −ve turners	**0.396**	**0.011**
		STI + ve turners	**0.570**	**0.009**
		STI −ve turners	0.106	0.658
Motility	Aggression	STI + ve & −ve turners	**0.363**	**0.021**
		STI + ve turners	0.281	0.230
		STI −ve turners	0.252	0.283
Turn angle 1 d post-6 fights	Turn angle 6 d post-6 fights	LTI^w/o^ winners & losers	**0.788**	** < 0.001**
		LTI^w/o^ winners	0.211	0.371
		LTI^w/o^ losers	**0.762**	**< 0.001**

*r* gives Pearson’s correlation coefficient and *p* the significance of difference. Statistically significant correlations are indicated in boldface.

As given in Table 1, pooled data of positive and negative turners also show that individual turning responses correlated positively with motility (total distance moved in 3 min: *r* = 0.396, *p* = 0.011, *n* = 40) and also with individual aggressiveness in a subsequent fight against a hyper-aggressive opponent (*r* = 0.363, *p* = 0.021, *n* = 40). However, separate analyses revealed only a correlation of turn angle with motility for positive turners (*r* = 0.570, *p* = 0.009, *n* = 20).

To check for possible effects of a longer isolation period in STI, an additional group was also taken as mature adults from the breeding colony, but isolated for 7 days. However, we found no significant differences in their responses to the touch stimulus compared to the STI group isolated for only 2 days. Before fighting, the prospective winners turned towards the stimulus and prospective losers away (means: + 24°, respectively −59°, significantly different to each other: *p* < 0.001, *n* = 15 each, but not to 2-day isolates: prospective winners and losers *p* = 0.457, respectively *p* = 0.789). Further, after fighting the mean turn angles did not change significantly (winners and losers compared to prospective winners and losers: *p* = 0.604, respectively *p* = 0.257).

### Long term isolates with adult contact as nymphs only (LTI^w/^)

**Figure 3:**
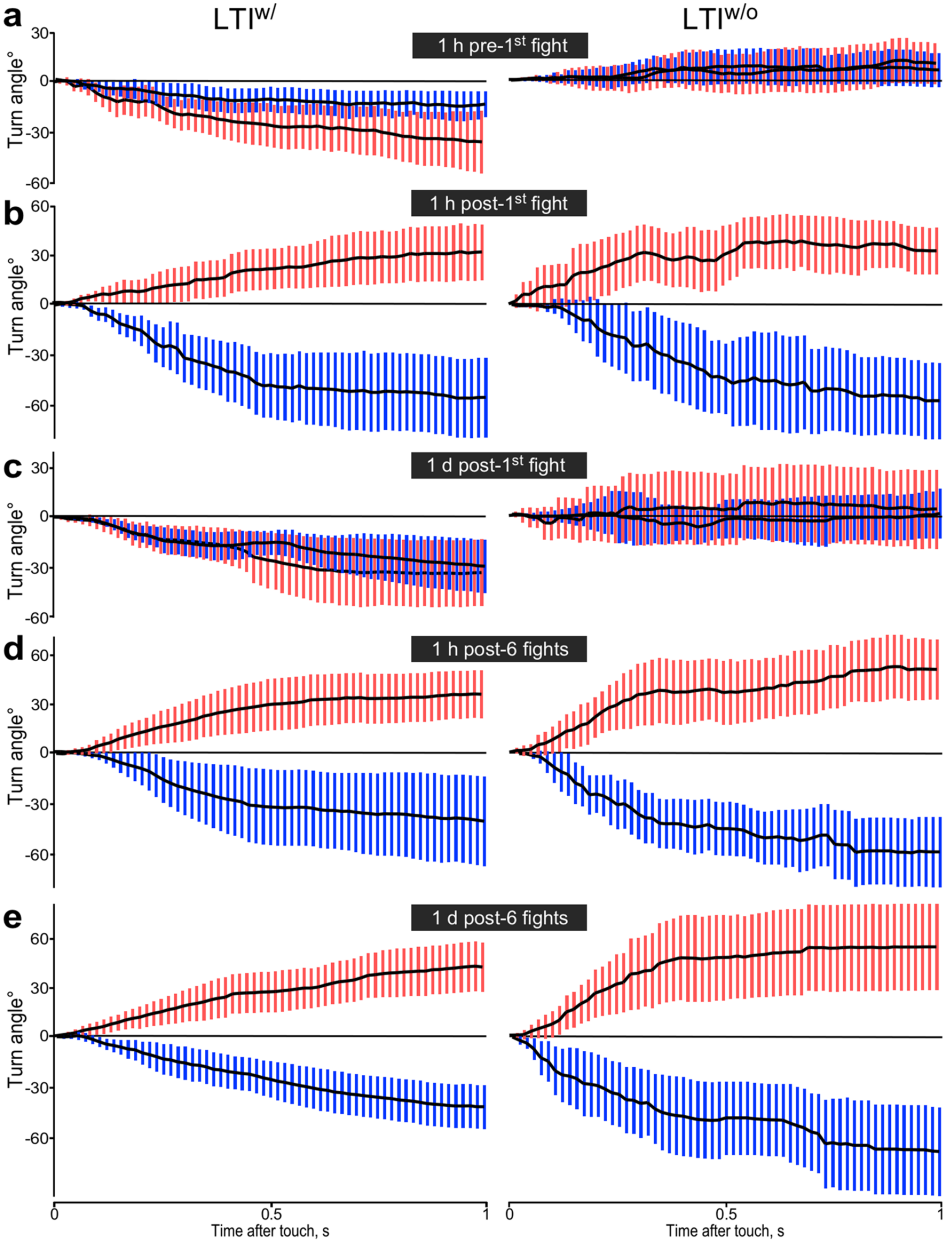
Turning responses of long term isolates with and without prior contact to adults (LTI^w/^, LTI^w/o^). Sequential plots of mean turn angles (black lines) for every frame with 95% CI. (**a**) Mature adults before fights (red and blue bars: prospective winners and losers). (**b**–**e**) Same animals as in (**a**) after various experiences: (**b**) 1 h after first fight, (**c**) 1 day later, (**d**) 1 h after 6 additional fights, (**e**) 1 day after the 6 fights (*n* = 20 for each). All components of the figures were created with canvas DRAW 5 for MAC (Version 5.0.2, ACD Systems, https://www.acdsystems.com/index).

**Figure 4:**
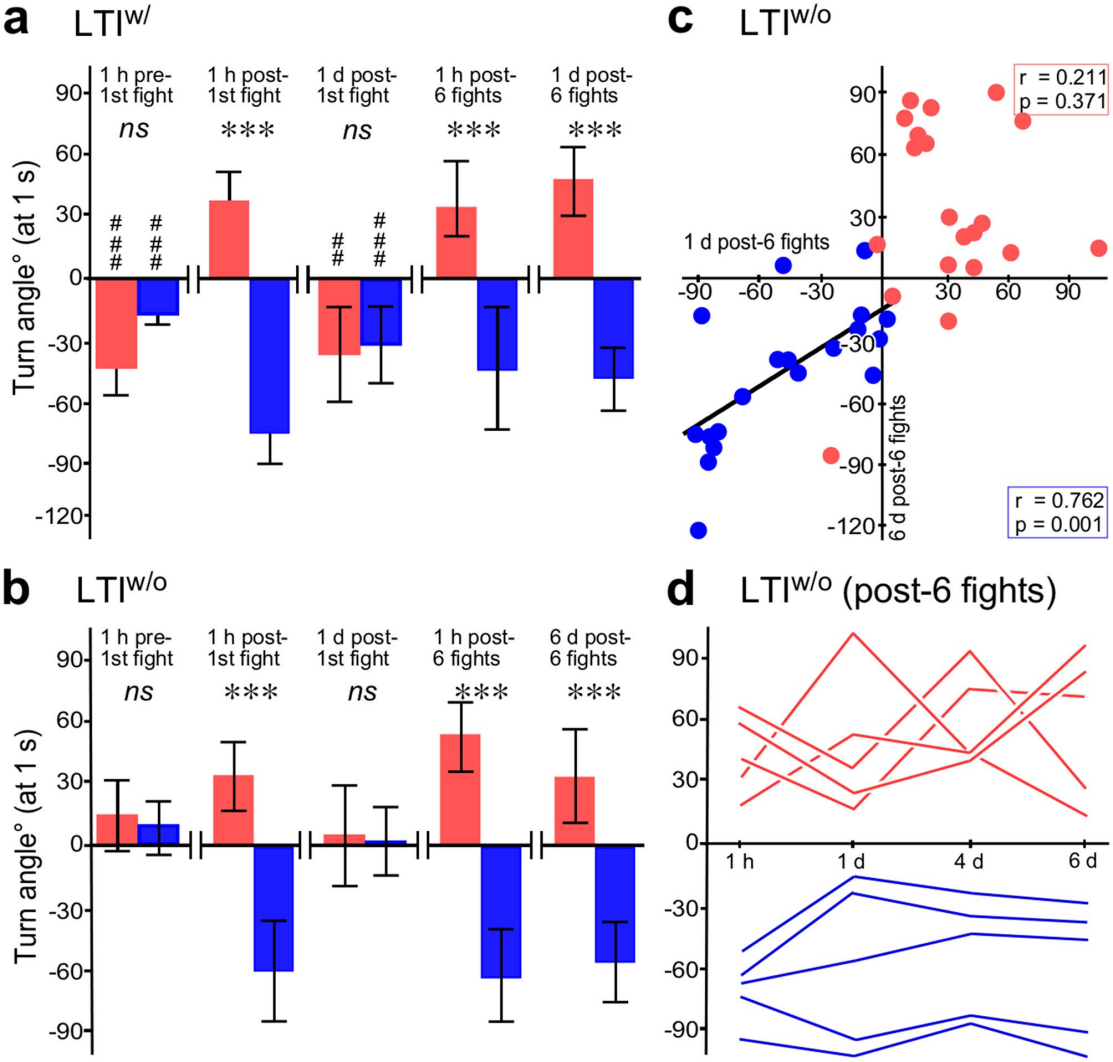
Turn angles for long term isolates 1 s after stimulus. (**a**,**b**) Box plots for LTI^w/^ respectively LTI^w/o^ crickets (red and blue bars: means for winners and losers respectively, whiskers: 95% CI). Asterisks indicate differences between groups and hashtags differences to zero: *ns* not significant, *** *p* < 0.001; # *p* < 0.025, ### *p* < 0.0005 (Bonferroni corrected alpha for 3 comparisons = 0.017). Note: the last bar chart in (**b**) is for data 6 days after 6 fights, in contrast to (**a**). (**c**) Plots of individual turn angles on day 1 *versus* day 6 after 6 fights for LTI^w/o^ winners and losers (red and blue circles respectively, *n* = 20 each). (**d**) Sequential plots of mean turn angles at different time points over a period of 6 days following 6 fights for randomly selected winners and losers (red and blue lines respectively, *n* = 5 each). All components of the figures were created with canvas DRAW 5 for MAC (Version 5.0.2, ACD Systems, https://www.acdsystems.com/index).

The turning responses of LTI^w/^ crickets are depicted as sequential plots in Fig. 3 (left side), and in Fig. 4a as box-whisker plots 1 s after the touch stimulus. Before their first fight, nearly all prospective winners and prospective losers showed negative turns in response to antennal stimulation (Fig. 3a), resulting in mean turn angles of -35° for the prospective winners and − 19° for the prospective losers 1 s after the touch stimulus (Fig. 4a; means significantly different to zero: *p* < 0.001, but not different to each other: *p* = 0.113, *n* = 20 each). However, 1 h after their very first fight, 80% of the resultant winners now turned towards the stimulus, whereas 90% of the losers turned away (Figs. 3b and 4a; means: winners = 32°, losers = − 55°, difference significant: *p* < 0.001, *n* = 20 each).

On the following day, winners and losers again both showed negative turning responses (Figs. 3c and 4a; means: winners = − 33°, losers = − 28°; both significantly different to zero: *p* = 0.001 and < 0.001 respectively; but not to each other: *p* = 0.666, *n* = 20 each). Immediately after this, we re-matched the same animals 6 times in succession to generate sixfold winners and sixfold losers. One hour after the 6^th^ fight the winners again showed approach behaviour and the losers avoidance (Fig. 3d; means: winners = 36°, losers = −39°, difference significant: *p* < 0.001, *n* = 20 each)**.** In contrast to one day after a single fight, this difference between multiple winners and losers was still evident on the following day (Fig. 3e; means: winners = 41°, losers = − 44°, difference significant: *p* < 0.001, *n* = 20 each).

### Long term isolates without prior adult contact (LTI^w/o^)

In marked contrast to LTI^w/^ prospective winners and losers, which had prior contact to adults and both of which turned away from stimulus, the LTI^w/o^ crickets showed no clear directional preference (Fig. 3a, right side, mean turn angles statistically different to LTI^w/^ for prospective winners *p* = 0.0002 and prospective losers *p* = 0.0013 with a significance level alpha for 3 comparisons of 0.017). Prospective losers had turned on average + 8° and prospective winners + 13° measured after 1 s (Fig. 4b; means not significantly different to each other: *p* = 0.602; both not significantly different to zero: winners *p* = 0.104, losers *p* = 0.210, *n* = 20 each). Even though LTI^w/o^ make comparatively small turns, we checked if individuals that turned > 10° towards the stimulus have a greater chance of winning a subsequent fight than those that turned > 10° away, but found none (+ ve turners > 10°: *n* = 18 of which 10 won; − ve turners > 10°: *n* = 9, of which 4 won; Fisher’s exact test *p* = 0.695). After this first fight, the LTI^w/o^ winners then turned towards the stimulus side, whereas LTI^w/o^ losers turned away (Fig. 3b; difference between groups after 1 s significant: *p* < 0.001). Despite this, on the following day, LTI^w/o^ crickets again showed on average no preferred turn direction as observed before fighting (data for winners and losers not significantly different to each other: *p* = 0.803 or to zero: winners *p* = 0.718, losers *p* = 0.951, but significantly different to the respective LTI^w/^ cohorts at this time: winners *p* = 0.014, losers *p* = 0.012 with a significance level alpha for 3 comparisons of 0.017). After 6 fights however, LTI^w/o^ winners consistently turned towards the stimulus, and the losers away from it over a period of 6 days (Fig. 4b; day 6 turn angles winners: mean = 33°, losers: mean = − 57°, difference significant: *p* < 0.001, *n* = 20 each). A plot of each individual’s turn angle 1 day after 6 fights versus 6 days after the contests revealed a linear correlation for losers, but not for winners (Fig. 4c; winners: *r* = 0.211, *p* = 0.371; losers: *r* = 0.762, *p* < 0.001, *n* = 20 each; Table 1). To further test for consistency, we took 5 randomly selected winners and losers of the six consecutive contests and plotted their turn angles at 4 different times after fighting which revealed consistency in the angular magnitude of the turning responses for losers, but again not for winners (Fig. 4d).

## Discussion

Insects provide unique systems to investigate the influence of early life experience in shaping behaviour^49^. Our study provides experimental evidence that consistent inter-individual differences in behaviour of adult male crickets can result alone from social experience gathered during nymphal development. Our first experiments with male crickets that were isolated individually for 48 h (STI) revealed that winners of a shortly preceding single fight mostly turn towards a touch stimulus directed at one antenna using the severed antenna of another male, whereas losers turn away (Fig. 2d). Similarly, dominant crickets also tend to turn towards a stimulus directed at one cercus^50^ (see also on crayfish^35,51^) and emerge quicker from a refuge than subordinates^25^. Here, however, we found that the difference in turning responses were already evident prior to fighting in the majority of the prospective winners and losers (Fig. 2c). Antennal stimulation occurs naturally when conspecific males first meet and takes the form of antennal fencing behaviour, where individuals lash each other’s antennae for about 2 s at 20 Hz^41^. During this, mechanical and pheromonal signals are transferred that act to release aggressive behaviour^41,48^. Although repeated stroking of an antenna with another male´s antenna nearly always evokes the mandible threat display^33,48^, this was never observed following a single, brief stimulus employed here. However, positive turners occasionally responded with a forward lunge, body jerks or the rival song, as often shown by males during aggressive interactions^44^. Since positive turners also have a predisposition to become dominants (90% win chance), the directional response is a good indicator of aggressive motivation, and as such can be considered as an element of the aggressive behavioural repertoire.

The results of our analysis of turning responses exhibited by STI crickets corroborates our earlier finding that male crickets that emerged as winners of a knock-out tournament are generally more active than the losers, both before and after fighting^19^. Apparently, therefore, adult male crickets taken from a standard breeding colony already show distinct ethotypes, that are not readily altered by a single agonistic experience: a proactive type, that is more active, more likely to approach a novel stimulus and more aggressive with a greater likelihood of becoming dominant than others of a reactive type, destined to be subordinate. It remains, however, to be tested whether these seemingly stable ethotypes can be eroded by multiple dominant or subjugation experiences (see on *Teleogryllus*^26^).

Since events during pre-adult life have lasting influences on adult behaviour in various other animals (damselflies^52^, frogs^53^, zebrafish^54^), we hypothesised that the two ethotypes found in adult crickets result from social experiences gathered during early post-embryonic development. Supporting this idea, mature adults raised as nymphs together with adult males, but separated just before reaching adulthood (LTI^w/^), all showed avoidance responses to antennal stimulation with no difference between prospective dominants and subordinates (Fig. 3a, left side and Fig. 4a). This fits with the observation that individual behavioural differentiation is generally lower in adult male crickets isolated at their final moult and the suggestion that behavioural individuality may generally result from social interactions^55^. Interestingly, adult crickets raised as nymphs with other nymphs, but without any previous contact to adults (LTI^w/o^), also showed no discernible difference between prospective winners and losers, but, in contrast to LTI^w/^ adults, no preferred directional response to the touch stimulus (Fig. 3a, right side and Fig. 4b). The key question arising is whether these differences to STIs emerge as a general consequence of chronic social isolation, or represent a more specific effect of development in the absence of social conflict, as recently shown in Zebrafish^56^. Social isolation can influence development, leading to alterations in the processing of environmental and social stimuli with wide ranging behavioural consequences in vertebrates and invertebrates^39,40,57^. In cockroaches, which like crickets are non-eusocial insects, social isolation is reported to induce a behavioural syndrome, typified by stronger exploration-avoidance, reduced foraging and reduced social interactions^36^. The cause was thought to result from decreases in metabolic and developmental rates and disturbances in processing environmental and social stimuli, resulting in inadequate behavioural responses, in particular to antennal contacts with partners. In crickets, social isolation leads to heightened aggression and exploratory behaviour^30,33^. However, this was shown to be a direct consequence of recovery from social subjugation in the absence of dominant males, and thus an indirect effect of social isolation^30^. We propose that this also explains the lack of a clear directional response to antennal stimulation in adults raised in the complete absence of adult males during development (LTI^w/o^). Accordingly, avoidance responses in adults that had contact to adults, either as nymphs (LTI^w/^), or as young adults (STI), are the result of prior social subjugation by adult males. We exclude the possibility that adult behaviour is influenced to any great extent by contact to nymphs during development. First, the population density of nymphs during rearing, and hence interactions between them, has no lasting effect on exploratory behaviour or aggression in adult crickets^10^. Second, in our previous study we found that nymphal crickets do not generate, or respond to adult male or female odour signals^33^. We also found that nymphs do not show physical aggression towards mature adults or other nymphs, and do not generate clear winners and losers, so that their social interactions were without long-term consequences for subsequent behaviour^33^. Notwithstanding this, nymphs treated with the aggression promoting octopamine agonist chlordimeform can show heightened aggression^33^, and nymphs have also been reported to acquire winner and loser effects lasting 5 min after interacting with a freshly moulted, immature adults^58^, which do not initiate fighting or exhibit physical aggression^43,59^. Nonetheless, this has little bearing on our interpretation, since the effects were very short lived and also not reported for interactions between nymphs and mature adults. However, nymphs of both sexes are frequently attacked by adult males (about once per hour), but not by adult females or other nymphs in the breeding colony^33^. We also discount the possibility that social isolation has a detrimental effect on perceiving the antennal stimulus. First, adults isolated for 2 and 7 days did not differ in their responses. Second, after either winning or losing a single fight, long term isolates (LTI^w/o^ and LTI^w/^) subsequently showed approach, respectively avoidance behaviour, equal to that of STI winners and losers. This is contrary to what would be expected if isolation had any permanent detrimental effects on sensory perception. Hence, while social isolation may have a predominantly detrimental effect on brain and behaviour in eusocial animals, this does not appear to apply to territorial animals, such as crickets, that invest in aggression to secure key resources. The difference in turning response established by one fight in LTI was no longer evident on the following day. This most likely occurs because the influence of neuromodulators mediating the behavioural effects of single wins and defeats, wane within about 3 h^29,30^. Nonetheless, after 6 successive defeats, at a frequency matching that of adult attacks on nymphs during development (once per hour), both groups of LTI crickets subsequently showed approach, respectively avoidance turns for at least as long as the 6 days tested (Fig. 4b). Although numerous authors have noted that dominant crickets tend to be both more aggressive and generally proactive in their behavior^17,19,23,25–27,33^, this is the first demonstration of a causal relationship. We conclude that experiencing repeated subjugation or dominance during development establishes stable proactive/reactive ethotypes.

We also analysed whether crickets show consistent inter-individual turning responses across time, indicating a “personality” trait, and checked for correlation between each individual’s turning response and its aggressive persistence as evidence for a “behavioural syndrome”^1,2,4,5^. With respect to the response to antennal stimulation, we found a good correlation between turn angles for STI crickets before and after a single fight when social status was disregarded, but not for separate analyses of the data for losers and winners, indicating consistent differences between the two ethotypes, but not necessarily between individuals (Fig. 2e). We suspect that correlation may be confuted by ambiguous designation of actual social status of the few winners that avoided the stimulus and the few losers that approached it. In weight-matched, single dyadic contests as practiced here, the winner is determined by the opponent’s actions^16,60^, and chances are it could have lost against a different opponent. Furthermore, similar to the finding that predator stress in crickets dissociates the correlation between consecutive measures of individual “boldness”^27^, we found that consistency of individual turning responses was influenced by fighting. Thus, a comparison of the turn angles of STI crickets on 2 consecutive days without an interposing fight, revealed a positive correlation for individuals showing avoidance, although not for those showing approach behaviour (−ve and + ve turners; Fig. 2f). Confirming this, longitudinal data gathered after multiple encounters revealed that inter-individual differences in turn angles were consistent in losers for 6 days after the last fight, though again not for winners (Fig. 4c,d). We suspect that the lack of correlation for successive measures of turn angle magnitude in winners and positive turners is a reflection of their more complex and variable response to the stimulus. For example, a greater variability in turn angles for individual positive turners could arise because they occasionally interrupt their turns with a forward lunge of minimal angular deviation, to halt in the near vicinity of the stimulus site and generate body jerks or sing the rival song. We conclude, with respect to subordinates at least, that crickets show consistent inter-individual differences in their behavioural response to a novel stimulus.

In addition to our present findings, numerous other studies have noted that dominant crickets tend to be more aggressive and exploratory in the face of novelty^19,25,50^, which suggests the existence of a “proactive–reactive” behavioural syndrome^1,5^. However, the critical test for a syndrome is whether a correlation between two or more behaviours for a group of individuals is significantly different from zero, whereby a larger correlation coefficient reflects tighter relationships^61^. Notably, while positive correlations between mating, exploratory and anti-predatory behaviour have been documented, e.g. in house crickets (*Acheta domesticus*^17^), no correlation was found between aggression and general activity or exploratory behaviour for this species^17^, *G. bimaculatus*^19^ or *Gryllus integer*^27^. In these studies, however, aggression was evaluated from the occurrence and/or duration of escalating agonistic behaviours during dyadic interactions between weight-matched individuals. As explained above, this is problematic, since the focal individual’s decisions to escalate or de-escalate and retreat is largely determined by the opponent^16,60^. However, using a multivariate mixed-effect model, positive associations were revealed between activity, exploration and measures of aggression for both contestants, and thus strong evidence for a proactive–reactive syndrome in *G. campestris*^23^. In our study, we opted to remove a source of within-individual variation by standardising one opponent (as suggested by Briffa et al*.*^16^). Individual focal animals were pitted against opponents made hyper-aggressive by flying^46^, from which they always retreated, so that the time persisting in fighting could be taken as a measure of individual aggressiveness. Our analysis then revealed positive associations between individual turning responses and aggression (Fig. 2g), as well as between activity and aggression (at least when taking all STI crickets into consideration, but not in all cases when positive and negative turners were analysed separately; see Table 1). Where significant, the correlation coefficients varied from 0.40 to 0.60, indicating a medium to large effect size^62^. Ideally, however, to qualify as a behavioural syndrome^61^, correlation should be demonstrated between independent behaviours, and shown to persist over a longer time period. This is difficult to achieve in our study, since aggression and the turning response to antennal stimulation were found to be related and interdependent behaviours. Even so, our data lend additional support for the hypothesis of a proactive–reactive syndrome in *G. bimaculatus* males, and indicate that this can arise solely as a direct consequence of aggressive experience. Individual adults that experience social adversity during nymphal or early adult life are more likely to show reduced aggression, social avoidance, lower motility and be less exploratory than adults that actively dominated others, without social retribution.

In mammals including humans, early life social adversity and stress throughout the juvenile period induces manifold behavioural, physiological, hormonal and neurochemical changes^63^, linked to numerous pathological conditions^39^. Mounting evidence suggest that behavioural syndromes and pathologies coupled to adversity are controlled by neuromodulators such as serotonin, noradrenaline and dopamine systems that control aggression and the drive to withdraw or approach in mammals^64,65^. Corresponding neurotransmitters in insects are known to bias the decision to retreat or approach a conspecific^66^ or a novel stimulus^67,68^. Investigations of the plastic responses of crickets to touch stimuli may, therefore, open a window into understanding the neuronal basis of behavioural individuality and syndromes. Particularly so, since the decision to approach or avoid is taken, surprisingly rapidly, within less than 35 ms. This leaves practically no time for complex integrative processing in higher brain centres. Turns are thus most likely initiated by a set of 4 fast conducting, giant descending interneurones in the brain, which transmit information from antennal mechanoreceptors to the thoracic motor centres^69^ to influence stepping and turning behaviour^70^. Future studies are now needed to demonstrate whether neurotransmitters that control aggression influence descending brain neurones and mediate the effects of social experience in establishing the proactive–reactive behavioural ethotypes described here.

## Data Availability

All data generated and analysed during the current study are fully available on reasonable request from the corresponding author.
